# The CDC Healthy Aging Research Network: Advancing Science toward Action and Policy for the Evidence-Based Health Promotion Movement

**DOI:** 10.3389/fpubh.2014.00261

**Published:** 2015-04-27

**Authors:** Basia Belza, Mary Altpeter, Steven P. Hooker, Gwen Moni

**Affiliations:** ^1^Department of Health Services, Health Promotion Research Center, University of Washington, Seattle, WA, USA; ^2^Department of Biobehavioral Nursing and Health Systems, Health Promotion Research Center, University of Washington, Seattle, WA, USA; ^3^Center for Health Promotion and Disease Prevention, University of North Carolina at Chapel Hill, Chapel Hill, NC, USA; ^4^School of Nutrition and Health Promotion, Arizona State University, Phoenix, AZ, USA

**Keywords:** evidence-based, dissemination, aging, networks, health promotion

Despite recent progress in the uptake of evidence-based health promotion (EBHP) programs within communities, many factors contribute to the need to focus on dissemination. These include the growth in the aging population, health care resource limitations, and interests in preserving community-based opportunities for maintaining independence and maximizing quality of life. For these reasons, The Prevention Research Centers’ Healthy Aging Research Network (HAN), funded by the Centers for Disease Control and Prevention’s (CDC’s) Healthy Aging Program, has as its core mission, to translate effective healthy aging interventions into sustainable community-based programs. Researchers and community-based stakeholders collaborate across HAN’s seven member center and two affiliate universities (Figure 1) to develop and implement health promotion programs for older adults at individual, organizational, environmental, and policy levels (1–3). This commentary highlights selected HAN contributions to the EBHP movement from 2001 to 2014. These contributions serve as examples of potential models for future partnership efforts to enhance implementation, dissemination, and sustainability of EBHP programs.

**Figure 1:**
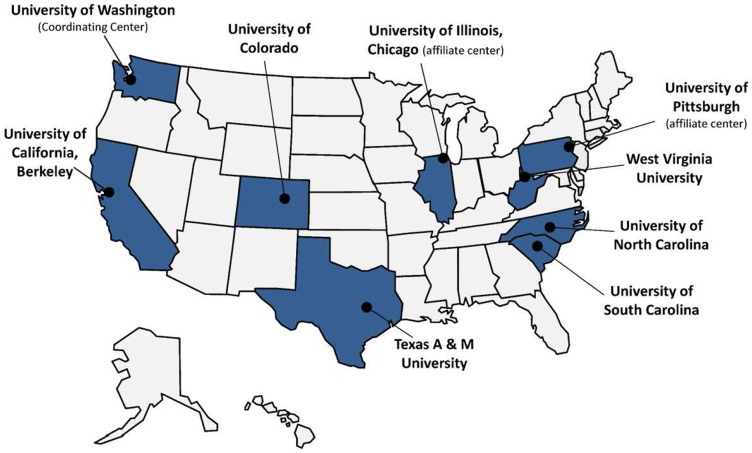
**CDC Healthy Aging Research Network (HAN) member centers and affiliates (FY 2009-2014)**.

## We Build the Foundation for EBHP Programs

The HAN has engaged researchers and practitioners from multiple disciplines and community organizations. We use the principles of community-based participatory research in diverse communities to develop research priorities (4–10) and to build a knowledge base for EBHP programs (11–14). Through these partnerships, HAN researchers have developed and tested practical tools and resources for the development, implementation and evaluation of interventions and frameworks (e.g., RE-AIM) for their dissemination and sustainability (15, 16).

For instance, HAN created and tested many of the programs described in this issue of *Frontiers* [i.e., EnhanceFitness, Chronic Disease Self-management Program (CDMSP), Fit and Strong! and Program to Encourage Active Rewarding Lives (PEARLS) (17–23)]. Nationally, we provided technical assistance on EBHP program implementation and evaluation for the Administration on Aging (within the Administration for Community Living) and grantee organizations. We have documented our methods of technical assistance in numerous peer-reviewed publications (2, 13, 19, 24, 25).

An example of our regional efforts is reflected in the HAN’s EBHP partnership with the Health Foundation of South Florida (HFSF) and Florida Healthy Aging Collaborative. HFSF is a not-for-profit grant-making organization with a focus on expanding access to affordable, quality healthcare for underserved populations in Florida’s Broward, Miami-Dade, and Monroe counties. HFSF launched a tri-county, 5-year $7.6 million health promotion and disease prevention initiative. HAN assisted with the initial planning and design of the initiative (e.g., program selection, evaluation components), helped launch workshops for prospective grantees, incorporated RE-AIM into the grant proposal structure, reviewed grant applications, and provided training materials for staff and grantees about RE-AIM. HAN also served on the leadership council and provided grantees with post-award technical assistance.

## We Enhance Capacity

To support the translation of EBHP programs into practice and policy, HAN has helped to enhance the capacity of researchers and practitioners. At the local, state, and national level, HAN has mentored and provided leadership opportunities for graduate students, early career investigators, and CDC Healthy Aging Program fellows by encouraging them to actively participate in HAN EBHP initiatives. Working with practice partners and national stakeholders, HAN has also built professional capacity by developing and delivering accessible, state-of-the-science trainings and resources. These include: conferences, online training modules (www.healthyagingprograms.org/content), monographs, and issue briefs about EBHP practice and various aspects of program delivery and quality assurance, physical activity, mental health, environment, and policy (26, 27).

Healthy Aging Research Network secured and leveraged a CDC conference grant to develop and deliver research-to-practice symposia on physical activity, mental health, and environmental policies. This series brought together national research and community partners to strategize how best to disseminate and sustain effective community-based programs and practices. For this series, HAN engaged new partners (e.g., AARP, The Carter Presidential Center, and the Rosalynn Carter Georgia Mental Health Forum, CDC Healthy Communities Program). HAN also secured additional funds from the Retirement Research Foundation to develop post-conference products and from the Agency for Healthcare Quality and Research to provide technical assistance. The ultimate result was the dissemination and uptake of a monograph (26), two coordinated and well-attended series of online webinars, as well as presentations and action briefs. HAN also contributed to the training of practitioners through presentations to the National Association of Chronic Disease Directors and National Association of State Units on Aging – Healthy Aging Initiative.

## We Affect Practice and Policy

At the national level, the Task Force on Community Preventive Services published recommendations from a HAN investigator-led review of community-based depression interventions on *The Community Guide* (28–32). This was the first time the Task Force accepted the findings of an “external” review. At the state level, HAN worked with the Washington State Unit on Aging to apply the recommendations to the agency’s depression screening policy to utilize a validated depression screening measure in annual assessments of clients who receive services. As a result, the Area Agencies on Aging in Washington have a better understanding of what proportion of their clients are depressed. In addition, practitioners can use this screening measure to determine client eligibility for PEARLS, an evidence-based program for depression. Consequently, evidence-based procedures and programs are now integrated into this state’s existing aging and social services.

In summary, HAN is the go-to source for technical assistance in large-scale EBHP program and policy design, implementation, and evaluation efforts with regional, national, and academic partners. HAN has harnessed the power and cost-effectiveness of multi-disciplinary, multi-site endeavors and become a recognized leader, able to convene disparate groups of stakeholders to build the science for EBHP. HAN investigators will continue to serve as facilitators and bridge builders to expand the overall public health and aging network within and outside of academia. Going forward, HAN investigators will continue to conduct EBHP research to improve capacity building, determine optimal methods for facilitating systems change in health promotion for older adults, and investigate the effectiveness of EBHP programs.

## Conflict of Interest Statement

The authors declare that the research was conducted in the absence of any commercial or financial relationships that could be construed as a potential conflict of interest.

This paper is included in the Research Topic, “Evidence-Based Programming for Older Adults.” This Research Topic received partial funding from multiple government and private organizations/agencies; however, the views, findings, and conclusions in these articles are those of the authors and do not necessarily represent the official position of these organizations/agencies. All papers published in the Research Topic received peer review from members of the Frontiers in Public Health (Public Health Education and Promotion section) panel of Review Editors. Because this Research Topic represents work closely associated with a nationwide evidence-based movement in the US, many of the authors and/or Review Editors may have worked together previously in some fashion. Review Editors were purposively selected based on their expertise with evaluation and/or evidence-based programming for older adults. Review Editors were independent of named authors on any given article published in this volume.
